# WIN 55,212-2, Agonist of Cannabinoid Receptors, Prevents Amyloid β_1-42_ Effects on Astrocytes in Primary Culture

**DOI:** 10.1371/journal.pone.0122843

**Published:** 2015-04-13

**Authors:** Diana Aguirre-Rueda, Sol Guerra-Ojeda, Martin Aldasoro, Antonio Iradi, Elena Obrador, Maria D. Mauricio, Jose Mª Vila, Patricia Marchio, Soraya L. Valles

**Affiliations:** Department of Physiology, School of Medicine, University of Valencia, Valencia, Spain; National Institutes of Health, UNITED STATES

## Abstract

Alzheimer´s disease (AD), a neurodegenerative illness involving synaptic dysfunction with extracellular accumulation of Aβ_1-42_ toxic peptide, glial activation, inflammatory response and oxidative stress, can lead to neuronal death. Endogenous cannabinoid system is implicated in physiological and physiopathological events in central nervous system (CNS), and changes in this system are related to many human diseases, including AD. However, studies on the effects of cannabinoids on astrocytes functions are scarce. In primary cultured astrocytes we studied cellular viability using MTT assay. Inflammatory and oxidative stress mediators were determined by ELISA and Western-blot techniques both in the presence and absence of Aβ_1-42_ peptide. Effects of WIN 55,212-2 (a synthetic cannabinoid) on cell viability, inflammatory mediators and oxidative stress were also determined. Aβ_1-42_ diminished astrocytes viability, increased TNF-α and IL-1β levels and p-65, COX-2 and iNOS protein expression while decreased PPAR-γ and antioxidant enzyme Cu/Zn SOD. WIN 55,212-2 pretreatment prevents all effects elicited by Aβ_1-42_. Furthermore, cannabinoid WIN 55,212-2 also increased cell viability and PPAR-γ expression in control astrocytes. In conclusion cannabinoid WIN 55,212-2 increases cell viability and anti-inflammatory response in cultured astrocytes. Moreover, WIN 55,212-2 increases expression of anti-oxidant Cu/Zn SOD and is able to prevent inflammation induced by Aβ_1-42_ in cultured astrocytes. Further studies would be needed to assess the possible beneficial effects of cannabinoids in Alzheimer's disease patients.

## Introduction

Alzheimer’s disease (AD) is a common neurodegenerative disease implicated in the aging process, affecting nearly 50% of people over 75 [1,2]. It involves neurofibrillary degeneration, extracellular accumulation of beta-amyloid peptide (Aβ) and synaptic dysfunction, resulting in neural cell death in the hippocampus and cerebral cortex, and in activation of glial cells [3,4]. Aβ can interact with different cellular components producing Ca^2+^ deregulation, oxidative stress and inflammation [5,6].

Astrocytes are specialized neural cells serving as a structural and metabolic support and trophic help to the brain [7]. Astrocytes also release cytokines and chemokines involved both in protective and toxic roles in neuroinflammatory processes [8]. However, released cytokines in neuroinflammation may induce deleterious effects on the viability and functionality of astrocytes [9]. Furthermore, in pathological situations such as hypoxia, cytokines induce activation of vascular endothelial cells thereby modulating inflammatory responses [10]. In AD, astrocytes are found around senile plaques producing phagocytosis, and cleaning up toxic compounds such as Aβ [11]. Moreover, when stimulated with compounds such as genistein or estradiol, astrocytes block the release of pro-inflammatory mediators and induce the synthesis of anti-inflammatory proteins [12].

Endocannabinoids have been implicated in various physiopathological events in different organs, including the peripheral and central nervous system (CNS) [13], and changes in the endocannabinoid system have been related to many human diseases, such as metabolic syndrome [14], neurodegeneration [15], inflammatory diseases [16], psychiatric disorders [17] and cancer [18]. The endocannabinoid signaling system is composed of anandamide and 2-arachidonoyl glycerol interacting with CB1 and CB2 cannabinoid receptors. Receptor signaling may involve mechanisms such as adenylyl cyclase blockade or activation of mitogen-activated protein kinases or ceramide signaling [13].

Different authors have proposed cannabinoids as preventive treatment in AD [19] due to their anti-inflammatory and neuroprotective properties [16]. In this sense, cannabinoids prevented microglial activation and cognitive impairment in Aβ-treated rats [19]. In mice exposed to Aβ, cannabinoids also suppress neuroinflammation by inhibiting inducible nitric oxide synthase (iNOS) expression and interleukin-1β generation [20]. However, the effects of cannabinoids on astrocytes functions have been poorly investigated. Therefore, we investigated the role of WIN 55,212–2 (WIN) as a neuroprotective agent against lesions induced by Aβ_1–42_ on cultured astrocytes.

## Material and Methods

### Materials

Dulbecco’s modified Eagle’s medium (DMEM) and fetal bovine serum (FBS) were obtained from Gibco (Gibco Invitrogen Corporation, Barcelona, Spain). The oligomers Aβ (40–1 and 1–42), were prepared following manufacture instructions (Sigma-Aldrich biotechnology). Briefly, the peptides were dissolved in H_2_O, and, for assembly the oligomers, preparations were heated for 24 h at 37ºC. WIN and 3-(4,5-dimethyl-2-thiazolyl)-2,5-dipheniyl-2H-tetrazolium bromide (MTT) were purchased from Sigma Chemical Co. (St Louis, MO). Enzyme-linked immunosorbent assay (ELISA) kits for interleukin 1β (IL-1β) and tumor necrosis factor (TNF-α) from Pierce Biotechnology, Inc. (Rockford, USA). Western Blot Chemiluminescent Detection System (ECL) was from Amersham (Amersham Biosciences, Barcelona, Spain). Monoclonal anti-peroxisome proliferator-activated receptor antibody (PPAR-γ) (1:250) and polyclonal anti-cyclooxigenase-2 antibody (COX-2) (1:250) from Sigma Aldrich (Madrid, Spain). Monoclonal p65 antibody (p65) (1:250) and monoclonal anti-Mn superoxide dismutase antibody (Mn-SOD) (1:250) from Santa Cruz Biotechnology (Madrid, Spain). Polyclonal anti-Cu/Zn superoxide dismutase antibody (Cu/Zn SOD) (1:250) from Assay Designs (Madrid, Spain). Monoclonal inducible nitric oxide synthetize (iNOS) (1:250) and anti-tubuline (1:1000) antibodies from Cell Signaling (Beverly, MA, USA). All other reagents are analytical or culture grade purity.

### Primary culture of cortical astrocytes

All animals were handled according to the recommendations of the Bioethics Committee of the School of Medicine of the University of Valencia, Spain. Ethics committee specifically approved this study. Cortical astrocytes were isolated from rat fetuses of 21 days gestation. Fetuses were obtained by cesarean section and decapitated. Cerebral cortices were removed and triturated 10–15 times through a Pasteur pipette with 10 ml DMEM. The cell suspension was filtered through nylon mesh with a pore size of 90 μm and re-suspended in DMEM containing 20% fetal bovine serum (FBS), supplemented with L-glutamine (1%), HEPES (10 mM), fungizone (1%), and antibiotics (1%). Cells were plated on T75 culture flask and maintained in a humidified atmosphere of 5% CO2/95% air at 37°C during 15 days. After 4 days of culture, the FBS was maintained at 20% and after 1 week of culture, the FBS content was reduced to 10%, and the medium was changed twice a week. The purity of astrocytes was assessed by immunofluorescence using anti-glial fibrillary acidic protein (astrocyte marker; Sigma-Aldrich), anti-CD-68 (microglial marker; Serotec), anti-myelin basic protein (olygodendroglial marker; Sigma-Aldrich) and anti-microtubule-associated protein 2 (neuronal marker; Sigma-Aldrich). The astrocyte cultures were found to be at least 99% glial fibrillary acidic protein positive. No cells were found to express CD-68, myelin basic protein, or microtubule-associated protein-2.

### Cell treatments

Ten days after seeding, WIN (10 μM) was added to culture flasks. Twenty-four hours later, 10 μM Aβ_1–42_ (toxic peptide) or Aβ_40–1_ (control peptide) (Sigma-Aldrich) were added to the flasks. Aβ_1–42_ concentration used in our study is in the range of toxic concentrations of the peptide [21,22]. Before incubation, the peptides were diluted in 100 μM of phosphate-buffered saline (PBS) and incubated for 24 h at 37º C. Assays were performed 24 h after peptide addition.

### MTT assay

Cell viability was determined by MTT assay. The MTT assay is a well-established, widely used and easily reproducible method for the assessment of cell viability and cytotoxicity [23,24]. Astrocytes were plated in 96-well culture plate and incubated with WIN during 24h. Subsequently, Aβ_40–1_ (control) and Aβ_1–42_ peptides were added to wells for another 24h. After cell treatments, the medium was removed and cells were incubated with red free medium and MTT solution [0.5 mg/ml, prepared in phosphate buffer saline (PBS) solution] for 4 h at 37ºC. Finally, the medium was removed and formazan particles were dissolved in dimethyl sulfoxide. Cell viability, defined as the relative amount of MTT reduction, was determined by spectrophotometry at 570 nm.

### Cytokine determination, IL-1 and TNFα

Astrocytes were seeded as previously published [12]. At the time of assay, the red phenol medium was removed and replaced by PBS containing 1 mg/ml bovine serum albumin, either in the presence or absence of Aβ_1–42_ (10 μM). IL-1β and TNF-α concentration (pg/ml) were ascertained using ELISA kits (Pierce Biotechnology, Inc.).

### Western blot analysis

Cultured cells were treated with lysis buffer and mechanically degraded to release the proteins. Protein concentration was determined using modified Lowry method [25]. Loading buffer (0.125 M Tris-HCl, pH 6.8, 2% SDS, 0.5% (v/v) 2-mercaptoethanol, 1% bromophenolblue and 19% glycerol) was added to protein sample and heated for 5 min at 95ºC. Proteins were separated on SDS-PAGE gels and transferred to nitrocellulose membranes in a humid environment using a transfer buffer (25mM Tris, 190mM glycine, 20% methanol). Membranes were blocked with 5% milk in TBS-T (0.05% Tween-20) and incubated with primary antibodies overnight at 4ºC. Membranes were washed 3 times with wash buffer TBS-T and incubated with a secondary anti-rabbit IgG or anti-mouse IgG (Cell Signalling Technologies Danvers, MA) antibody conjugated to the enzyme horseradish peroxidase (HRP) for 1 h. Membranes were washed three times and proteins were detected using the ECL method as specified by the manufacturer. Autoradiography signals were assessed using digital image system ImageQuant LAS 4000 (GE Healthcare).

### Statistical analyses

Values are expressed as mean±S.D. Differences between groups were assessed by one-way analysis of variance (ANOVA). Statistical significance was accepted at P ≤ 0.05. Data sets in which *F* was significant were examined by a modified *t*-test.

## Results

### Protective role of WIN on cell viability

**Fig 1:**
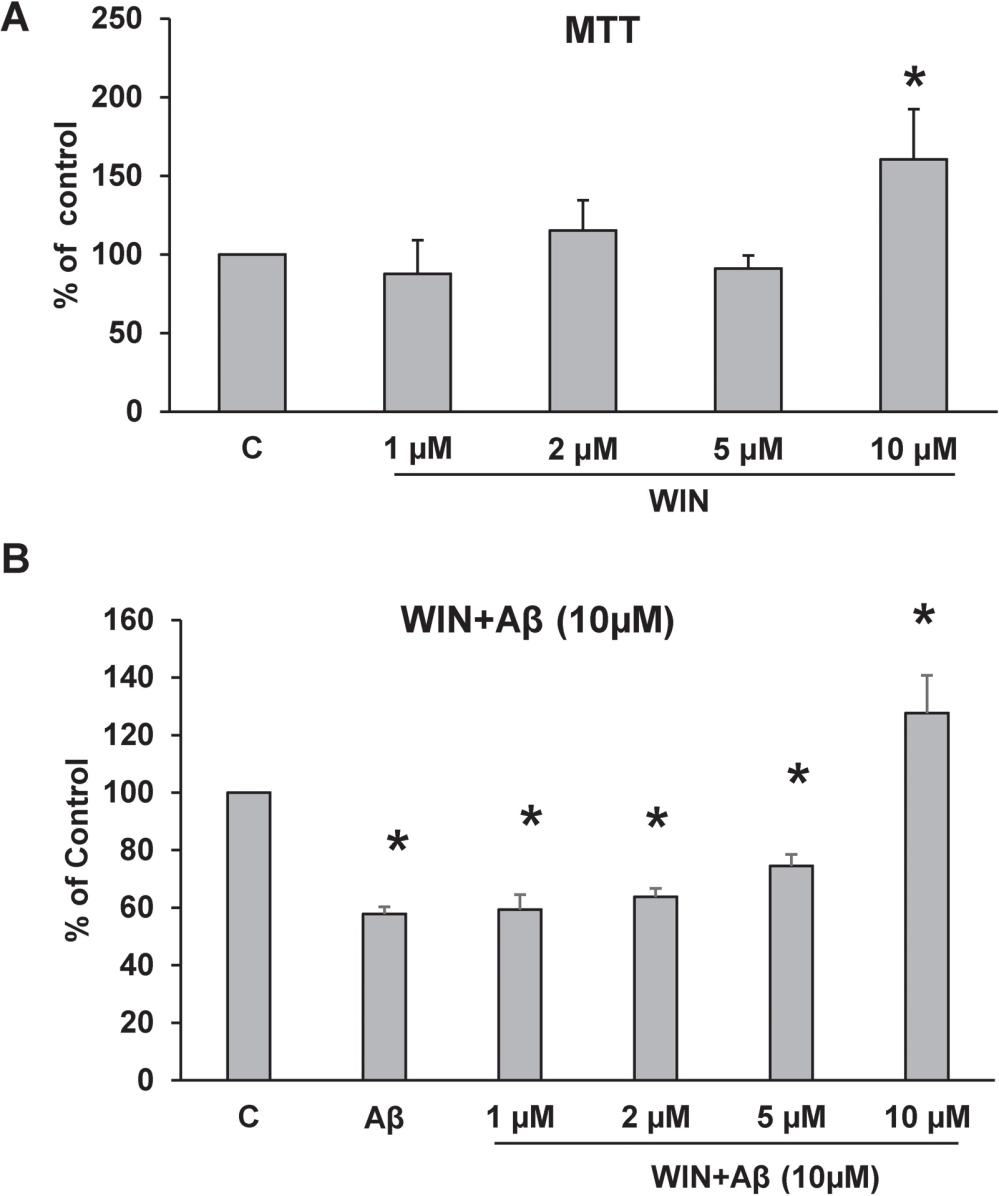
Astrocytes viability. (A) Astrocytes viability induced by WIN. Concentration-dependent viability of WIN (1, 2, 5, 10 μM) was determined by MTT assay for 24 h. Data are means ± SD for 4 independent experiments. *p<0.04 comparing WIN vs control cells. (B) Astrocytes viability in cells treated during 24 h with 10 μM Aβ_40–1_ (control peptide, C), 10 μM Aβ_1–42_ (Aβ) and WIN (1, 2, 5, 10 μM) + 10 μM Aβ_1–42_ (WIN + Aβ). Data are means ± SD of 3 independent experiments. *p<0.05 *vs* control cells.

The role of WIN on cell viability was studied using MTT conversion assay. Fig 1A shows that incubation with WIN at different concentrations induced a significant increase in cell viability at 10 μM. Consequently, that concentration was used in future experiments. Astrocytes previously incubated with 10 μM Aβ_1–42_ for 24 h significantly decreased cell viability compared to control cells (Fig 1B). Furthermore, pretreating astrocytes during 48 h with WIN (10 μM) prevented the decrease in cell viability induced by Aβ_1–42_ (WIN + Aβ), conversely WIN (1, 2, 5μM) did not have any effect (Fig 1B).

### WIN prevents IL-1β and TNF-α increase elicited by Aβ_1–42_

**Fig 2:**
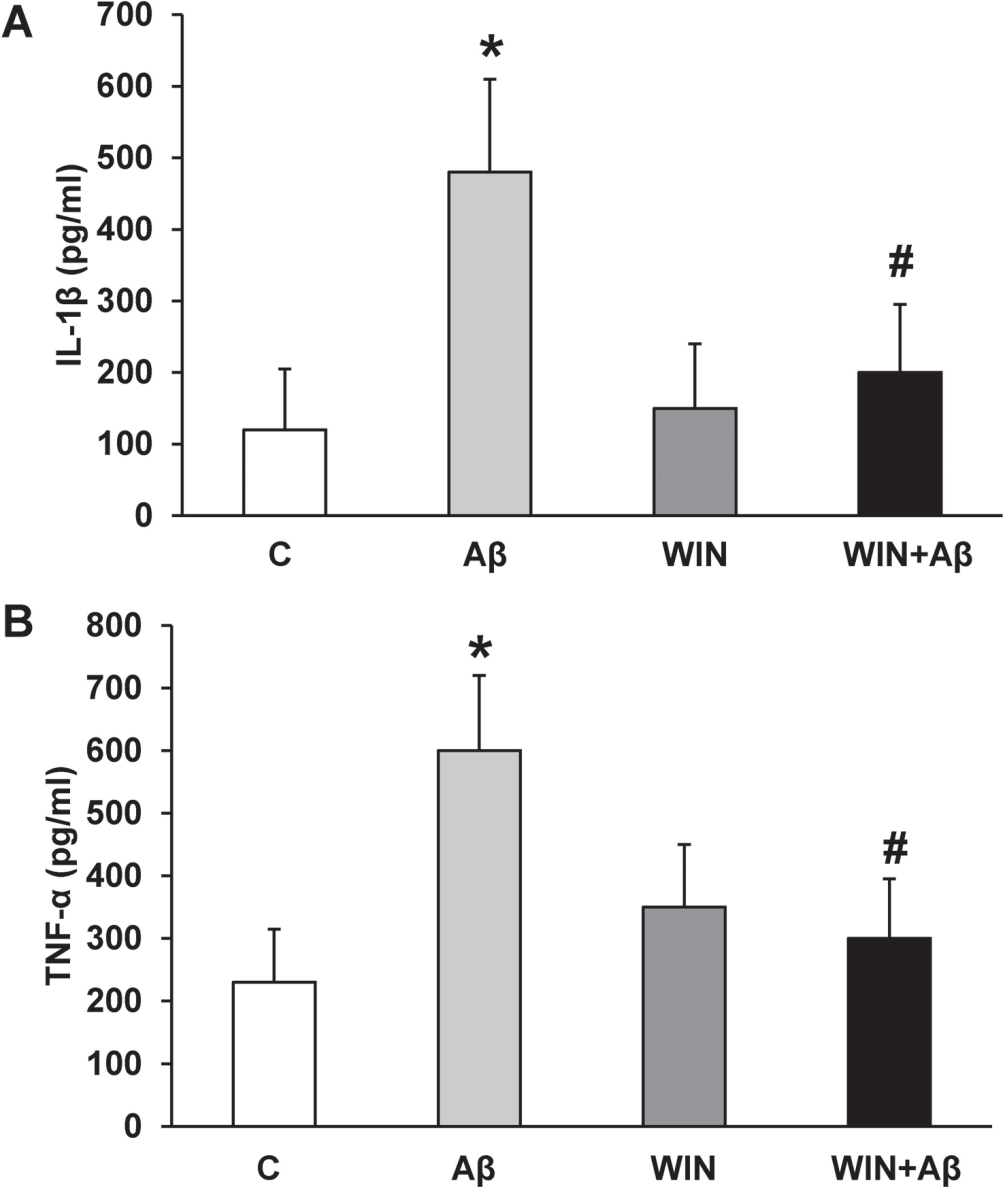
IL-1β and TNF-α secretion. WIN prevents the increase of IL-1β and TNF-α secretion caused by Aβ_1–42_ in astrocytes. Cells were incubated with 10 μM Aβ_40–1_ (control peptide, C), 10 μM Aβ_1–42_ (Aβ), 10 μM WIN + 10 μM control peptide (WIN) and 10 μM WIN + 10 μM Aβ_1–42_ (WIN + Aβ). Cell culture supernatants were harvested, and IL-1β (panel A) and TNF-α (panel B) secretion were determined by ELISA. Values are means ± SD of replicate experiments from 4 independent astrocytes cultures. *p<0.05 *vs* control astrocytes. #*p*<0.05 *vs* Aβ_1–42_ treated cells.

Cultured astrocytes were incubated with 10 μM Aβ_1–42_ and proinflammatory mediators TNF-α and IL-1β were detected by ELISA. Aβ_1–42_ increased 4.5-fold IL-1β release (480.4±150.3 pg/ml) compared with control (103.9±82.9 pg/ml) (Fig 2A) and 2.4 fold TNF-α release (605.3±103.4 pg/ml *vs* 210.5±85.3 pg/ml in control group) (Fig 2B). Furthermore, WIN pre-treatment (10 μM) prevented the increase in pro-inflammatory mediators induced by Aβ_1–42_ (Fig 2A and 2B).

### Effect of Aβ_1–42_ and WIN on p65 protein expression

**Fig 3:**
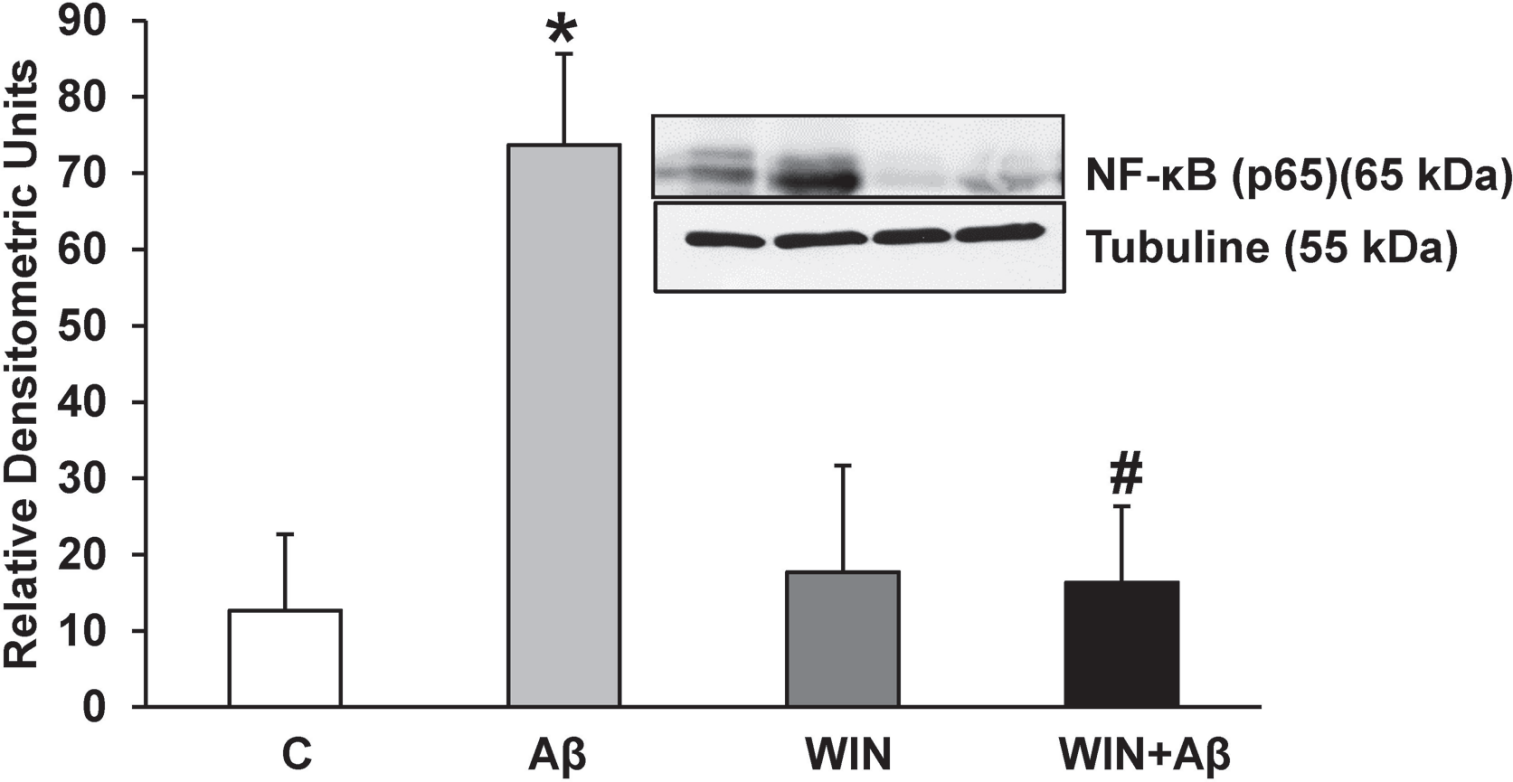
p65 protein expression. WIN 55, 212–2 prevents p65 expression induced by Aβ_1–42_ in astrocytes in primary culture. p65 and α-tubulin expressions were determined by Western-blot in astrocytes treated for 24 h with 10 μM Aβ_40–1_ (control peptide, C), 10 μM Aβ_1–42_ (Aβ), 10 μM WIN + 10 µM control peptide (WIN) and 10 μM WIN + 10 μM Aβ_1–42_ (WIN + Aβ). A representative immunoblot of each protein is shown and tubulin was used as control amount of protein. Data are means ± SD of 5 independent experiments. *p<0.05 *vs* control cells. #*p*<0.05 *vs* Aβ_1–42_.

Nuclear factor κB (NF-κB), the pro-inflammatory transcription factor is formed by different subunits. We measured p65 protein expression by western-blot. Incubation with Aβ_1–42_ increased p65 protein expression compared with control astrocytes (Fig 3), which was prevented by WIN pretreatment. (p<0.05 compared with Aβ_1–42_ treated astrocytes).

### WIN prevents COX-2 and iNOS protein increase induced by Aβ_1–42_ peptide

**Fig 4:**
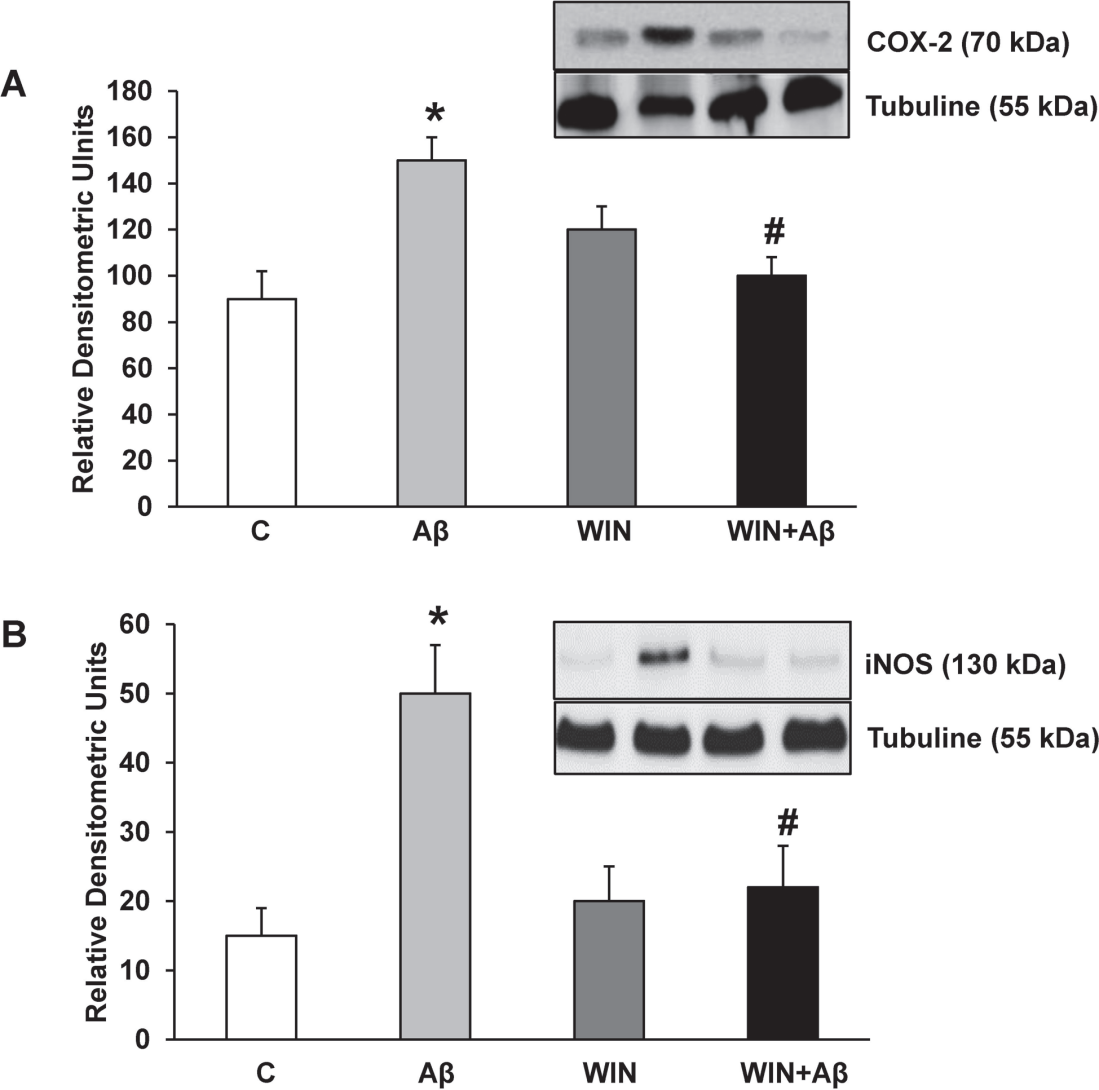
COX-2 and iNOS protein expression. WIN prevents COX-2 and iNOS expression induced by Aβ_1–42_. COX-2 (panel A), iNOS (panel B) and α-tubulin expressions were determined by Western-blot in astrocytes treated for 24 h with 10 μM Aβ_40–1_ (control peptide, C), 10 μM Aβ_1–42_ (Aβ), 10 μM WIN + 10 μM control peptide (WIN) and 10 μM WIN + 10 μM Aβ_1–42_ (WIN + Aβ). A representative immunoblot of each protein is shown and tubulin was used as control amount of protein. Data are means ± SD of 6 independent experiments. *p<0.05 *vs* control cells. #*p*<0.05 *vs* Aβ_1–42_.

Incubation with Aβ_1–42_ significantly increased inflammatory proteins COX-2 (Fig 4A) and iNOS (Fig 4B) expressions compared to control. Furthermore, pretreating astrocytes with WIN prevented the effects produced by Aβ_1–42_.

### Effect of Aβ_1–42_ and WIN on PPAR-γ protein expression

**Fig 5:**
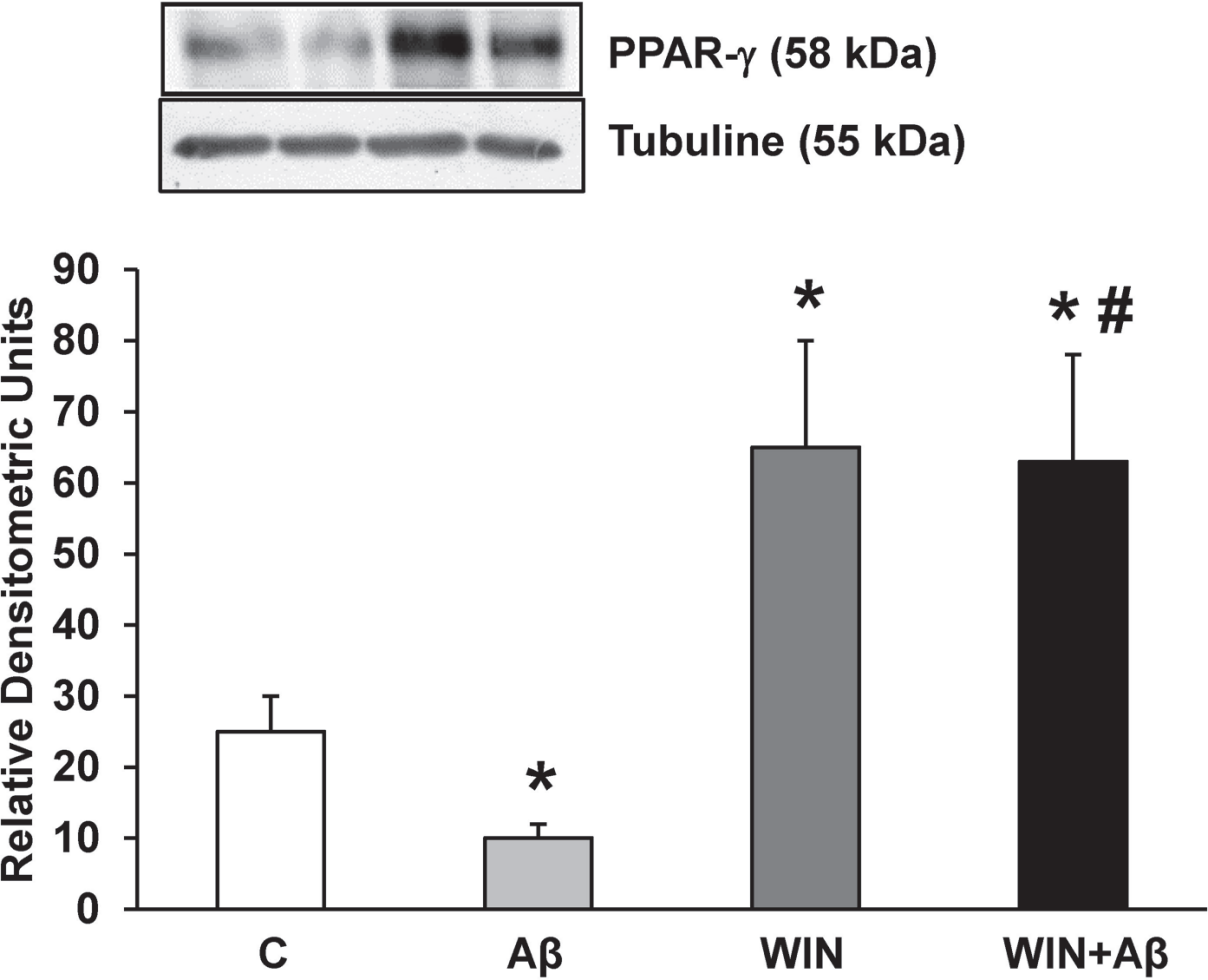
PPAR-γ protein expression. WIN induces PPAR-γ expression in astrocytes in primary culture treated with Aβ_1–42_. PPAR-γ and α-tubulin expressions were determined by Western-blot in astrocytes treated for 24 h with 10 μM Aβ_40–1_ (control peptide, C), 10 μM Aβ_1–42_ (Aβ), 10 μM WIN + 10 μM control peptide (WIN) and 10 μM WIN + 10 μM Aβ_1–42_ (WIN + Aβ). A representative immunoblot of each protein is shown and tubulin was used as control amount of protein. Data are means ± SD of 4 independent experiments. *p<0.05 *vs* control cells. #*p*<0.05 *vs* Aβ_1–42_.

Pro-inflammatory gene expression is downregulated by PPARs family [26]. We found that pretreatment with WIN (10 μM) increased PPAR-γ expression compared to control cells (Fig 5). Incubation with Aβ_1–42_ significantly decreased PPAR-γ expression that was prevented by WIN pretreatment.

### Effect of Aβ_1–42_ and WIN on Cu/Zn SOD and Mn SOD protein expression

**Fig 6:**
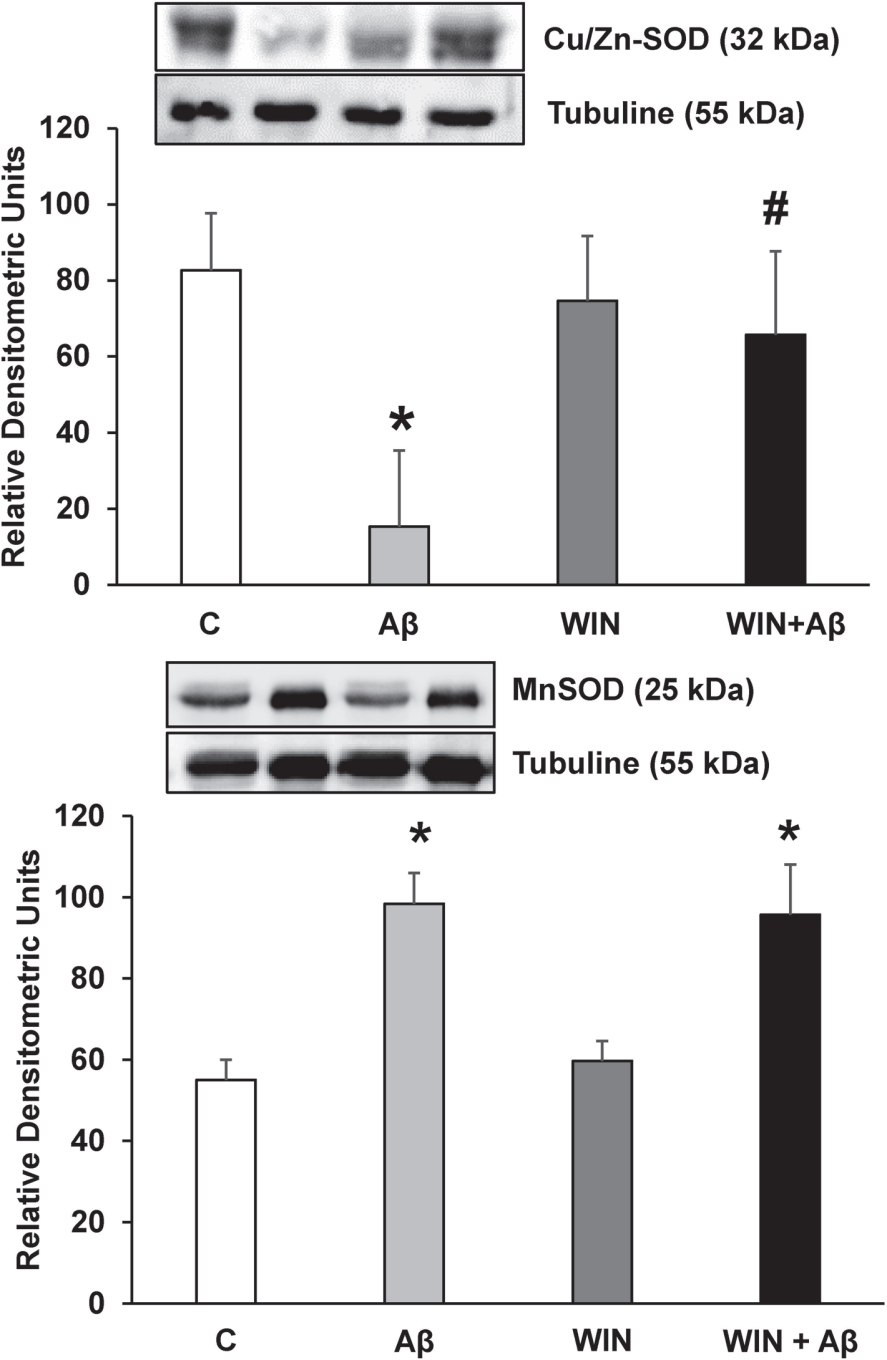
Cu/Zn-SOD and Mn-SOD protein expressions. WIN prevents Cu/Zn-SOD expression decrease in astrocytes in primary culture treated with Aβ_1–42_. Cu/Zn-SOD, Mn-SOD and α-tubulin expressions were determined by Western-blot in astrocytes treated for 24 h with 10 μM Aβ_40–1_ (control peptide, C), 10 μM Aβ_1–42_ (Aβ), 10 μM WIN + 10 μM control peptide (WIN) and 10 μM WIN + 10 μM Aβ_1–42_ (WIN + Aβ). A representative immunoblot of each protein is shown and tubulin was used as control amount of protein. Data are means ± SD of 4 independent experiments. *p<0.05 *vs* control cells. #*p*<0.05 *vs* Aβ_1–42_.

Superoxide dismutase is a key antioxidant enzyme. In our study, incubation with Aβ_1–42_ decreased Cu/Zn SOD expression in astrocytes in primary culture which was prevented by WIN pretreatment, evidencing that WIN could play a neuro-protective role against oxidative stress induced by Aβ_1–42_ peptide (Fig 6A). On the other hand, our results indicated that Mn SOD protein expression is increased in presence of Aβ_1–42_. Pretreatment with WIN did not prevent Mn SOD increase induced by Aβ_1–42_ (Fig 6B).

## Discussion

Oxidative stress and inflammation are the main mechanisms in the progression of various neurodegenerative diseases, including AD [27–30]. In our study, we determined different markers involved in inflammation and oxidative stress induced by the Aβ_1–42_ peptide in primary cultures of astrocytes, with the aim to assess the antioxidant and anti-inflammatory effects of cannabinoid WIN. We found that WIN significantly increased astrocytes viability compared to control cells. Furthermore, WIN prevented the decrease in astrocytes viability induced by Aβ_1–42_.

It has been shown that cannabinoids preserve neurons from Aβ exposure by activating MAP kinase cascade [31] and by anti-oxidative and anti-apoptotic effects [32]. Moreover, some studies demonstrated that cannabinoids protect glial cells from death [33,34]. Nevertheless, in cancer, where cells are highly proliferative and undifferentiated, treatment with cannabinoids can block cell proliferation in a dose dependent manner [35–38], demonstrating that the effects of cannabinoids on cell viability are probably dependent on cell type [39] and developmental stage [40].

Expression of CB1 [41] and CB2 [42] receptors in rat culture astrocytes have been published and also dual activation of both cannabinoid receptors by WIN (the mixed non-selective CB1/CB2 agonist) in rat cortical astrocytes have been detected [41] On the other hand, WIN confers its protective and anti-inflammatory effects against Aβ injury through both CB1 and CB2 receptors [43]. Given that our results there is expression of both types of cannabinoid receptors, CB1 and CB2, it is likely that the effect of WIN observed in our study is due to the interaction with both types of receptors, consistent with published results by Fakhfouri and cols [44].

We found that WIN prevented the increase of inflammatory mediators IL-1β, TNF-α, NF-κB, iNOS and COX-2, as well as the decrease of the anti-inflammatory mediator PPAR-γ induced by Aβ_1–42_ in astrocytes in primary culture. The inflammatory process is a characteristic mechanism in the development of AD, and pro-inflammatory agents are involved in the progression of cell damage [45,46,47,12]. Moreover, it is known that astrocytes participate in the inflammatory process induced by Aβ_1–42_ [27,28,48]. Initially, inflammation is beneficial since it produces pro-inflammatory substances involved in tissue protection, limiting the survival and proliferation of cells exposed to toxic agents, such as Aβ_1–42_ [49,50]. However, sustained inflammatory response could lead to neurotoxic damage or cell death [12,51,8]. NF-κB proteins are up-regulated in inflammation conditions such as astroglial activation induced by Aβ_1–42_ oligomers [52]. In this regard, we found an increase in NF-kB/p-65 expression in astrocytes after addition of Aβ_1–42_ that was prevented by WIN pretreatment. Valles and collaborators [53] found that the cytokine-receptor complex is able to bind to cytokines and other proteins of the extracellular matrix, producing inflammatory signals which could be important in pathologies such as Alzheimer's disease [53,54]. In agreement with our results, different authors have reported that cannabinoids mitigate neural cell activation in the neuroinflammatory response induced by Aβ_1–42_, reducing the levels of pro-inflammatory molecules such as IL-1β, TNF-α, COX-2 and iNOS [55,56,57]. Likewise, the activation of cannabinoid receptors diminishes the release of IL-1β, IL-6 and TNF-α in microglial cells [58,59,60] as well as COX-2 and iNOS [61]. Studies conducted in rats pretreated with the Aβ peptide found that WIN prevented cognitive impairment, glial activation and neuronal loss [19,62,63], and also reduced COX-2, iNOS and TNF-α levels [63,64].

Kainu et al. [65] demonstrated for the first time the presence of mRNA and protein PPAR-γ in CNS cells. Subsequent studies have detected PPAR-γ expression in microglial and astrocytic cells [66,12]. PPAR-γ agonists protect against Aβ-induced inflammatory and neuronal damage [67,68], thus making neurons and astrocytes potential therapeutic targets for PPAR-γ ligands [69,12]. Astrocytes also express the largest levels of PPAR-γ in the neural tissues [70,71]. As other authors [72,73,74], we found a decreased expression of PPAR-γ in astrocytes treated with Aβ_1–42_. Esposito et al. [56] showed in neurons that cannabinoids may act as neuro-protective agents by PPAR-γ activation. In this study, we demonstrate an increase in this protein expression in astrocytic cells previously incubated with WIN. Furthermore, we found for the first time that WIN prevents PPAR-γ expression decrease induced by Aβ_1–42_ peptide in astrocytes in primary culture. There is strong evidence to suggest that some cannabinoids can act on PPARs through either direct or indirect pathways. In order to directly act on nuclear transcriptional factors PPARs, exogenous cannabinoids need to pass through plasma membrane and be transported into nucleus which may involve certain membrane and intracellular transporters. However, we still cannot rule out that cannabinoids effects could be indirect through the binding of other cellular targets which in turn induces PPARs activation [75]. In fact, WIN attenuates amyloid-beta-induced neuroinflammation in rats through activation of cannabinoid receptors and PPAR-γ pathway [44].

**Fig 7:**
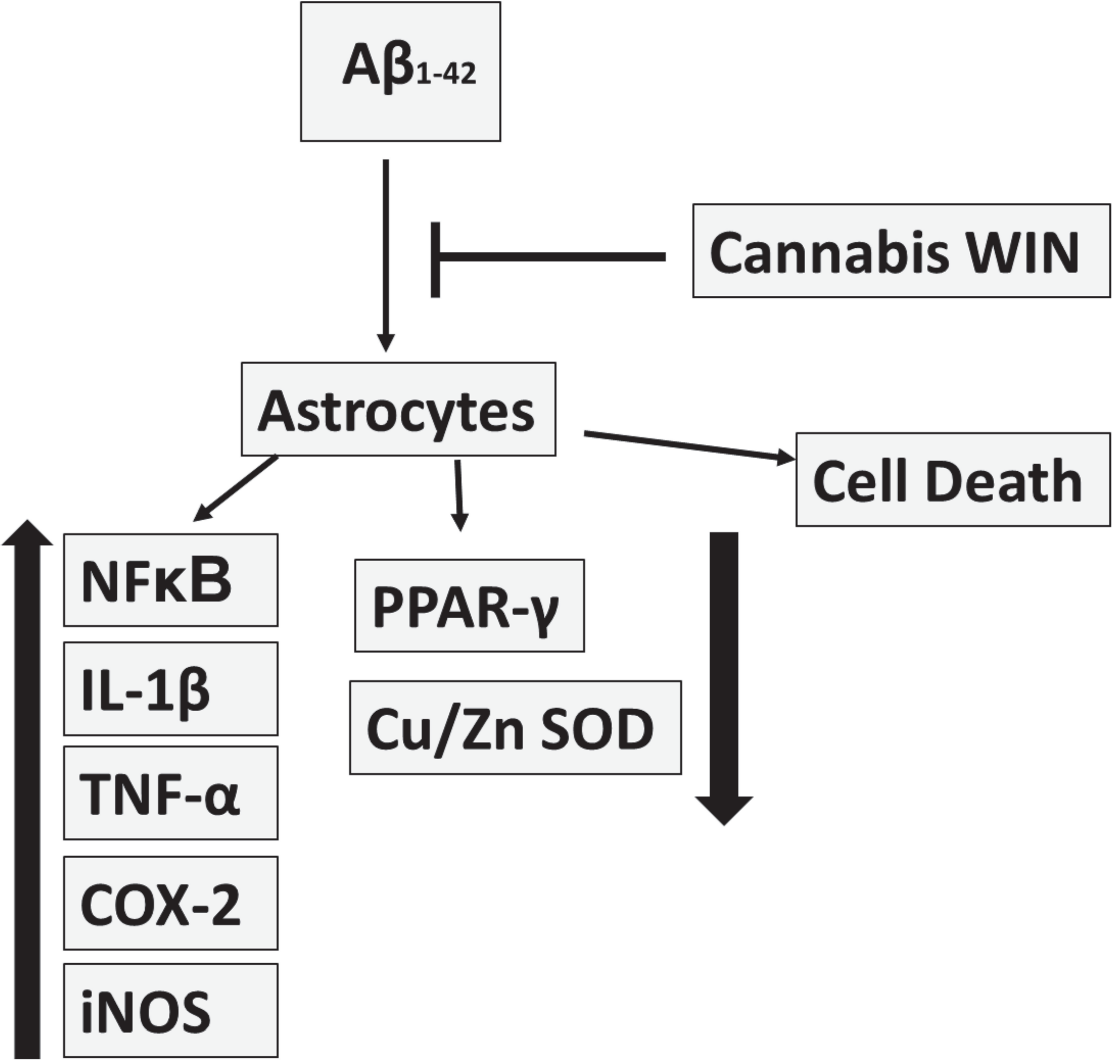
Preventive function of cannabinoid WIN on Aβ_1-42_-induced toxic effects in astrocytes in primary culture. Cannabinoid WIN 55,212–2 increases cell viability and anti-inflammatory response in cultured astrocytes and prevents inflammatory effects induced by Aβ_1–42_.

Different authors have demonstrated the role of oxidative stress in AD [76–79]. The cumulative damage caused by free radicals induces alterations in the activity or expression of antioxidant enzymes like catalase or SOD. These enzymes were found to be decreased in both CNS and peripheral tissues of AD patients [80,81]. In this sense, we demonstrate that Cu/Zn SOD is decreased in astrocytes treated with Aβ_1–42_. Our results are consistent with those reported by other authors, highlighting the role of oxidative stress in the development of AD [82]. New substances are under research to reduce damage caused by oxidative stress in this disease. Widely distributed in the body, cannabinoids receptors were discovered few decades ago and are still under research [57]. Few studies address the effect of cannabinoids on oxidative stress. For instance, cannabinoids were found to prevent or antagonize oxidative stress toxicity in cortical neurons in cultures [83,84], and in lymphoblastic cells [85]. Studies with PC12 cells exposed to Aβ_1–42_ peptide demonstrated that cannabinoids reduced reactive oxygen species production and membrane lipid oxidation [86,32]. Our results provide evidence that Aβ_1–42_ decreases Cu/Zn SOD expression in astrocytes in primary culture, and pretreatment with WIN increases Cu/Zn SOD expression, preventing the decrease caused by Aβ_1–42_. These findings indicate that cannabinoids could act as a protective agent against oxidative stress caused by Aβ_1–42_. In Fig 7 the set of results are summarized. However our results indicate that Aβ_1–42_ elevated Mn SOD protein expression, increasing mitochondrial biogenesis mechanism, such as we previously published [74]. Pretreatment with WIN did not prevent Mn SOD overexpression induced by Aβ_1–42._ Mn SOD plays a role in the adaptive response which protects brain cells from damage, as in the case of AD. In fact, Mn SOD preserves neurons against oxidative stress [87] and protects developing neurons from β-amyloid toxicity [88]. This enzyme catalyzes the conversion of superoxide radicals to molecular oxygen and H_2_O_2_, whereas glutathione peroxidase, peroxiredoxin reductase and catalase neutralize H_2_O_2_. Overexpression of Mn SOD induces cognitive recovery and reduces Aβ levels in AD animal models [89]. Furthermore, Mn SOD deficiency increases β-amyloid levels and amyloid plaque burden, promoting the development of behavioural disturbances [90].

Preclinical data suggest a beneficial role of some cannabinoids for treatment of different diseases. Dronabidol, an oil-based solution of Δ9-THC, is used as anti-emetic and appetite stimulant [91]. Δ9-THC also decreases agitation present in the advanced stage of AD [92]. In 2003, the FDA granted the patent for cannabinoids as antioxidants and neuro-protectants (U.S. Department of Health and Human Services). Despite these promising preliminary results, the clinical utility of cannabinoids in AD is still to be determined [93].

## Conclusions

Taken together, our findings show that cannabinoid WIN increases cell viability and anti-inflammatory response in astrocytes in primary culture and prevents cell death induced by Aβ_1–42_. Furthermore, WIN increases expression of anti-oxidant Cu/Zn SOD and is able to prevent inflammation induced by Aβ_1–42_ in astrocytes. In this sense, clinical studies are needed to evaluate the neuro-protective effects of cannabinoids in Alzheimer´s disease.
